# Abdominal pain during the menopause transition and early postmenopause: observations from the Seattle Midlife Women’s Health Study

**DOI:** 10.1186/s40695-019-0046-5

**Published:** 2019-08-02

**Authors:** Nini G. L. Callan, Ellen S. Mitchell, Margaret M. Heitkemper, Nancy F. Woods

**Affiliations:** 1 Helfgott Research Institute, National University of Natural medicine, Portland, OR USA; 2 Department of Family and Child Nursing, University of Washington, Seattle, WA USA; 3 Department of Biobehavioral Nursing and Health Informatics, University of Washington, Seattle, WA USA

**Keywords:** Menopause, Abdominal pain, Gastrointestinal, Anxiety, Stress, Hormones

## Abstract

**Objective:**

To assess the relationship between abdominal pain severity during the menopausal transition (MT) and age, MT stage, reproductive biomarkers, stress biomarkers, and stress perceptions.

**Methods:**

Women ages 35–55 were recruited from multiethnic neighborhoods in the greater Seattle area from 1990 to 1992, for an original study cohort of 508. From 1990 to 2013, a subset of this cohort consented to ongoing annual data collection by annual health questionnaire, health diary, and daily menstrual calendar. Beginning in 1997, a portion of these women also provided a first morning voided urine specimen to be assayed for levels of estrone glucuronide (E_1_G), follicle stimulating hormone (FSH), testosterone, cortisol, norepinephrine, and epinephrine. To identify how changes in abdominal pain severity changed over time in relation to age, MT stage, reproductive biomarkers, stress-related biomarkers, and stress-related perceptions, mixed effects modeling was used.

**Results:**

In a univariate model, E_1_G (*p* = 0.02) and testosterone (p = 0.02) were significantly and negatively related to abdominal pain severity, while perceived stress (*p* = 0.06), tension (*p* <  0.001), and anxiety (p <  0.001) were significantly and positively associated. In a multivariate model, increasing age (*p* = 0.001) and E_1_G (*p* = 0.04) were negatively associated with abdominal pain severity, and anxiety (p = 0.00) positively associated. Testosterone did not improve the fit to the final model, nor did tension or perceived stress.

**Conclusions:**

These results suggest that age, anxiety, and E_1_G each show a significant association with abdominal pain severity in the MT. In contrast, stress perception, tension, testosterone, stress biomarkers, and MT stage do not. These factors should be evaluated further in research on abdominal pain experienced during the MT and early postmenopause years.

## Introduction

In the United States (US), it is estimated that approximately 3 million women enter into the menopause transition (MT) each year [1]. The MT includes three stages—*Early Menopause Transition*, *Late Menopause Transition*, and *Early Postmenopause*—each of which is distinguished by progressive irregularity and eventual cessation of the menstrual cycle, outlined in detail elsewhere in this paper. Many physical and psychosocial symptoms can accompany this transition, the severity of which disrupts women’s quality of life (QOL) to varying degrees [2, 3]. A 2016 study revealed that of a representative sample of 3397 US women with an age range of 40–69 years, 52% would always prefer a reduced lifespan to experiencing menopausal symptoms at their worst for 30 days [4]. According to that study, the third least desirable menopausal symptom (i.e. third most troublesome and interfering with QOL)—and the focus of this current study—was abdominal pain (as measured by the Women’s Health Questionnaire).

Abdominal pain is a relatively common symptom in the general population, accounting for 11% of emergency department (ED) visits each year, and in ED patients older than 65 years, it is the third most common health complaint overall [5, 6]. In a representative sample of 2786 men and women ages 70–90, abdominal pain was found to be associated with the female sex, and interestingly, in both sexes it decreased significantly with age [7]. In a representative sample of 4581 Danish men and women of varying ages, abdominal pain occurred significantly more often among women compared to men (49%, versus 38%), resolved less frequently in women compared to men (31% versus 43%), and revealed a decrease in prevalence with advancing age, starting with highest prevalence occurring in the age 30 group and lowest prevalence occurring in the age 60 group for both women and men (52 and 48% versus 43 and 30%) [8]. These findings suggest sex- and age-specific etiological factors in the development of abdominal pain. However, it is unclear whether abdominal pain experienced in the MT is due to the normal aging process, or to the physiological changes specific to the MT (i.e. decreasing estrogen and progesterone due to follicular depletion in the ovaries, and increasing FSH.) [9, 10].

One study using data from the Study of Women’s Health Across the Nation, which included a representative cohort of 1495 women, provides compelling evidence to suggest the latter—that abdominal pain could be due to physiological changes specific to the MT. Bodily pain (as measured by the Short Form-36 index) increased steadily throughout the MT, and began decreasing steadily after the final menstrual period (FMP; this marks the occurrence of “menopause”, and thus, the beginning of the postmenopausal years) [11]. These findings suggest that pain experienced during the MT may be specific to the distinct physiologic changes occurring throughout that transition and not to the general process of aging. However, it is unknown whether or not this trend of decreasing general pain extends to abdominal pain experienced in the MT.

Regarding sex-specific contributions to the development of abdominal pain in the MT, many studies show a clear role for female sex hormones in the etiology and pathophysiology of several pain-focused disorders (lower back pain, joint pain, musculoskeletal pain, and genitourinary pain) [12–14]. One systematic review of fluctuating hormone levels and gastrointestinal (GI) symptoms in women with and without Irritable Bowel Syndrome (IBS) revealed that there was an increase in GI symptoms—including abdominal pain—around the early MT, when ovarian hormones are beginning to fluctuate, suggestive of a hormonal contribution in the etiology of GI symptoms, and specifically abdominal pain, at that stage of life [15]. At this point, more research is needed to determine if fluctuating or declining hormone levels are, in fact, etiological to abdominal pain experienced throughout the MT.

In addition to sex- and age-specific factors, research suggests that stress and anxiety may also play a role in the experience of abdominal pain. A study involving reproductive aged women found that pelvic pain was associated with anxiety (as measured by the General Anxiety Disorder-7 questionnaire) and abdominal pain [16]. Another study suggested that abdominal wall pain is associated with central sensitization, which would predispose one to abdominal pain, and yet another study found that chronic abdominal pain was associated with lower urine cortisol levels [17, 18]. These potential relationships are currently unexplored in an MT population.

The proposed relationships described above have begun to be explored by investigators for the Seattle Midlife Women’s Health Study (SMWHS), a longitudinal, prospective study that followed a cohort of women from 1990 to 2013, as they transitioned from the late reproductive phase of life through the MT and into their postmenopausal years. Urinary biomarkers were collected several times each year starting in 1996 and continued until 2005 (e.g. estrone glucuronide [E_1_G], follicle stimulating hormone [FSH], testosterone, cortisol, norepinephrine, and epinephrine), as well as an annual health questionnaire, menstrual calendar, and health questionnaire. Details of the study are described elsewhere [19]. Previous research has revealed a general pattern of estrogen decrease and FSH increase throughout the MT, which was corroborated by SWMHS findings [20, 21]. The SMWHS also revealed that throughout the MT, cortisol levels increased over time, and these increases were associated with E_1_G, testosterone, FSH, norepinephrine, and epinephrine [20–22]. To date, no research has been done assessing the possible associations between these hormones and abdominal pain experienced throughout the MT.

The purpose of this study was to begin addressing the gap in knowledge regarding abdominal pain experienced by women transitioning from their late reproductive years, through the MT, and into the early postmenopausal years, by elucidating the effects of several covariates on abdominal pain severity throughout the MT. These covariates included: menopausal transition stage (Late reproductive, Early MT, Late MT, and Early Postmenopause [PM]), reproductive hormone biomarkers (E_1_G, FSH, testosterone), stress-related biomarkers (cortisol, epinephrine, and norepinephrine), and stress-related perceptions (anxiety, tension, and daily stress level).

## Methods

### Design and sampling

The SMWHS is a prospective, repeated-measures study conducted in the greater Seattle area from 1990 to 2013—the data used in this study were gathered throughout the 23 years of data collection. The study focused on the natural menopausal transition and the symptoms, stresses, and hormones associated with it, which is described in greater detail elsewhere [19]. Briefly, recruitment took place between 1990 and 1992 from a population-based sample. Inclusion criteria for study participation was comprised of the following: 35–55 years of age; in the late reproductive stage, or early or late menopause transition stages; had a period within the previous 12 months; had at least one ovary and an intact uterus; was not pregnant or lactating; and could read and speak English. Eligible recruits included 820 women, 508 of whom began the study and provided initial cross-sectional data. The longitudinal component of the study included annual data collection by daily menstrual calendar and annual health questionnaire, and 390 of the 508 women entered into this part of the study. A health diary was included in the longitudinal component of the study as an additional data collection tool, but only a subset of women chose to complete it. The health diary was collected on days five through seven of the menstrual cycle, each month from the beginning of the study until the year 2000, and quarterly from that time on (2001—2013). Questions in the health diary included a symptom checklist with severity scale, indicators of health behaviors, and perceptions of stress.

From 1996 to 2005, a subset of study participants (*N* = 170) agreed to also provide a monthly first-void urine specimen for biomarker analysis. Collection coincided with the health diary on day six of the menstrual cycle of each woman. If a woman was no longer experiencing a monthly period, she chose a day on which urine samples were to be collected that then remained consistent for all subsequent months.

Participants remained in the study up to 5 years postmenopause, at which point, they became ineligible for study participation. Over the course of the study, of the original study cohort (*N* = 508), 173 dropped out due to personal reasons, 173 became ineligible, and 162 were lost to contact. This current analysis includes a subset (291 participants) of the original cohort of 508 women, and were included in the current analysis because they contributed health diary data, and completed menstrual calendars, and thus their cycles could be classified into LR stage or one of the MT stages (see *MT Stages* below). In addition, these women did not meet any exclusion criteria: use of hormone replacement therapy, incomplete health diary entries, a hysterectomy, inadequate calendar data, or receiving chemotherapy or radiation therapy. Of this subset, 131 participants also provided urine samples to be assayed for reproductive and stress-related biomarkers.

### Measures

**Fig. 1:**
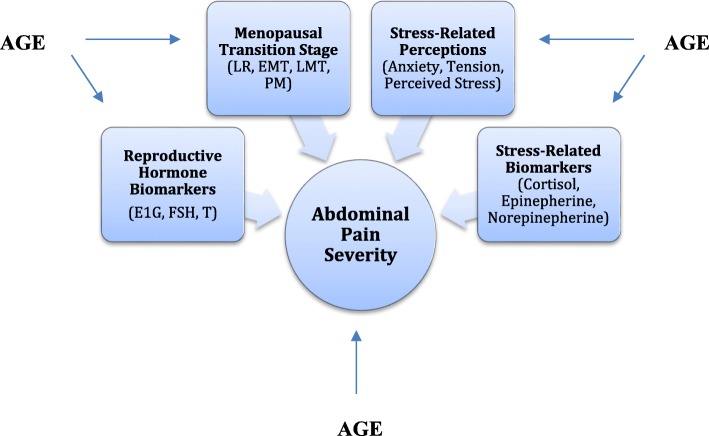
Model of the hypothesized relationships between predictor (Age), covariates, and outcome measure (abdominal pain severity)

The following measures were included in analyses presented here (see Fig. 1): MT stages, urinary assays (reproductive biomarkers and stress-related biomarkers), health diary data (stress-related perceptions), and the outcome measure of abdominal pain severity.

#### MT stages

Women were classified into reproductive aging stages throughout their involvement in the study, using menstrual calendar data. MT stages were defined using the staging criteria developed for the SMWHS by Mitchell, Woods, and Mariella, and validation of the stages came from the ReSTAGE collaboration [23–27]. The names of each stage matched the Stages of Reproductive Aging Workshop (STRAW) recommendations: *late reproductive, early MT, late MT*, and *early PM* [28]. The *late reproductive stage* was defined as the time during midlife when cycles were regular. *Early MT* was defined as persistent irregularity of more than 6 days’ absolute difference between any two consecutive menstrual cycles in a calendar year, as well as no skipped periods. *Late MT* was defined as the persistent skipping of one or more menstrual periods. Amenorrhea for 60 or more days in the calendar year constituted a skipped period, and ‘persistence’ was defined as a skipped period, irregular cycle, or event occurring one or more times in the 12 months subsequent to the initial occurrence of any one of those events. *Early PM* was defined as the 5 years after the FMP. FMP was retrospectively identified after 1 year of unexplained amenorrhea and was considered synonymous with the onset of menopause.

#### Urine sample collection and biomarker analysis

On day six of the menstrual cycle, first-voided morning urine specimens were collected. In the case of unidentifiable or erratic menstrual periods, or the complete cessation of menstrual periods, a consistent monthly date was used for collection timing. Women abstained from exercise, smoking, and caffeine use prior to urine collection. Sodium metabisulfite and sodium ethylenediaminetetraacetic acid were used to preserve the urine samples, which were then frozen at − 70 ° C.

All biomarkers were analyzed using urinary assays performed in our laboratories, and assays included a pooled in-house urine control, as well as a Bio-Rad Quantitative Urine control. A urine sample on the standard curve was repeated after every ten unknowns in order to monitor assay performance. Controls, specimens, and standards were tested in duplicate, and those with a coefficient of variance greater than 15% were repeated. Multiple samples from each participant were assayed in the same batch, batched per the year of collection; samples from a calendar year were generally assayed during the following calendar year. To correct for variations in urine concentration (as measured by specific gravity), endocrine hormone concentrations were expressed as a ratio to the concentration of urine in the sample from which they were taken.

Biomarkers assayed for included urinary E_1_G, follicle stimulating hormone (FSH), testosterone, cortisol, and the catecholamines epinephrine and norepinephrine. The coefficients of variance for each biomarker are as follows (intra-assay and inter-assay): E_1_G (2.1 and 9.6%), FSH (3.7 and 7.1%), testosterone (8.75 and 12.38%), cortisol (4.6% and 8.2–12.5%), epinephrine (4.7 and 7.85%), norepinephrine (4.7 and 7.85%). Further details of the assays for each respective biomarker are described elsewhere [22, 29–32].

#### Health diary data

##### Stress-related perceptions

*Anxiety, tension,* and *perceived stress* were assessed by questions posed in the health diary. *Anxiety* and *tension* were separately assessed by questions asking women how anxious or tense they felt, respectively, within the past 24 h. Answers were based on a scale of 0 to 4, where 0 was ‘absent’ and 4 was ‘extreme’. *Perceived stress* was assessed by the question: ‘How stressful was your day?’. Answers were based on a scale of 1 to 6, where 1 was ‘not at all’ and 6 was ‘extremely, a lot’. A significant correlation (*r* = 0.35, *p* <  0.01) between a global stress rating and the sum of stress ratings across multiple dimensions was found by Brantley et al. [33]

##### Outcome variables: symptom severity

The outcome variable was abdominal pain severity, within the past 24 h, as reported in the health diary (asked as, “*Please fill in the number that best describes how severe each item was for the past w4 hours.”).* Answers were given according to the same 0 to 4 scale described above. No other measures, such as frequency or duration, were asked.

### Analysis

To investigate abdominal pain severity and whether the predictor (age), and the covariates (MT stage, reproductive and stress biomarkers, and reported levels of tension, anxiety, and stress) had associations with it, multi-level modelling (MLM) using the R library was used, in a univariate analysis first, followed by a multi-variate analysis. Both random and mixed effects were used. These models are briefly described below, and in detail elsewhere [19, 34]. The use of MLM was justified because data were collected at multiple time points for each woman, over several years, throughout the duration of the study.

To determine the best fit for the data, two models were initially tested. The first model assumed a fixed effect—or, a single rate of change—for abdominal pain severity (i.e. abdominal pain would change at the same rate each year for all women). The second model assumed a random effect—or, an individual rate of change—for abdominal pain severity (i.e. abdominal pain severity would change at a different rate each year for each woman). For both models, the measure of time was tracked by *age* (the predictor), and the mean age (calculated to be 47.6 years) was used to improve the interpretability of the results (i.e. the results were centered on age). To assess which model was the best fit for the data, the maximum likelihood estimation, according to the Akaike Information Criterion (AIC), was used [35]. A significant AIC *p*-value (*p* <  0.05) would indicate that assuming a random effect was more appropriate than assuming a fixed effect. Analyses revealed that the random effects model was the best fit for the data (*p* <  0.001), and this model was then used for analysis of the data.

The best fitting model—the random effects model—was then used to analyze each covariate independently (i.e. a univariate analysis; see Table 2), to determine whether doing so was an improvement on the model including age as a measure of time. Covariates that revealed an improved model fit to the data in the univariate analysis were then simultaneously entered into and tested by the final model (i.e. a multivariate analysis; see Table 3). Results of the multivariate analysis were examined for consistency of direction with the univariate results. When effects differed in direction, the model was re-specified to eliminate variables with effects that indicated multicollinearity. When stress, anxiety, and tension were examined in the multivariate model, change of sign of the effect from positive to negative for stress and tension when included in the model with age and anxiety suggested multicollinearity. Thus, subsequent models were tested that included only anxiety, but not stress or tension. Also, the model was re-specified by removing testosterone from the biomarker indicators based on similar effect size to E_1_G in the univariate models, and also because of the findings that when both were included in the multivariate model, neither met our criterion for statistical significance.

Urine samples were not collected until year six of the study, which limited the number of women available for biomarker analyses to those willing to give regular urine samples and who were still involved in the study after 6 years. In addition, there were missing data for some of the covariates. Taken together, these factors resulted in covariate observation and sample size variability—the number of women represented in each covariate analysis ranged from *n* = 130 to *n* = 291, and the number of observations for each covariate ranged from *n* = 3325 to *n* = 6977.

## Results

Baseline characteristics for study participants included a mean age of 41.5 years (standard deviation [SD] = 4.3 years), 15.9 years of education (SD = 2.8 years), and a median family income of $38,200 (SD = $15,000). The majority of participants described themselves as White (82%) and were currently employed (87%). A much smaller proportion of participants described themselves as Asian-American (9%) or African-American (7%). Most were married or partnered (71%), and a smaller proportion divorced or widowed (22%), or were never married or partnered (7%). Statistically significant differences were seen between eligible and ineligible women in race/ethnicity, income, and years of education. Women included in the analyses had more formal education and higher income, and were more likely to describe themselves as White, than those who were ineligible for inclusion. These characteristics are displayed in more detail in Table 1.

**Table 1:** Sample characteristics at start of study (1990–1991) of the eligible and ineligible women in the mixed effects modeling analyses of abdominal pain severity

	Eligible Women (*n* = 291)	Ineligible Women (*n* = 217)	
Characteristic	Mean (SD)	Mean (SD)	*P*-value ^a^
Age (years)	41.5 (4.3)	42.0 (5.0)	0.180
Years of education	15.9 (2.8)	15.3 (3.0)	0.030
Family income ($)	38,200 (15,000) 18.6 (7.0)	35,200 (17,600) 17.1 (8.3)	0.040
Characteristic	N (Percent)	N (Percent)	*P*-value ^b^
Currently employed			0.400
Yes	254 (87.3)	184 (84.8)	
No	37 (12.7)	33 (15.2)	
Race/ethnicity			0.001
African American	20 (6.9)	38 (17.5)	
Asian /Pacific Islander	27 (9.3)	16 (7.4)	
White	238 (81.8)	153 (70.5)	
Other (Hispanic, Mixed)	6 (2.1)	10 (4.6)	
Marital Status			0.420
Married/partnered	278 (71.1)	141 (65.0)	
Divorced/widowed/not partnered	63 (21.7)	62 (28.6)	
Never married/partnered	21 (7.2)	14 (6.5)	

^a^ Independent t-test

^b^ Chi-square test

Statistical significance was set as *p* <  0.10 for the univariate analyses to screen covariates for inclusion in a final multivariate model. *Age* was not found to be a significant predictor for abdominal pain, nor were any of the MT stages. Of the reproductive aging biomarkers, *E*_*1*_*G* and *testosterone* were related to a statistically significant lower abdominal pain (beta coefficient = − 0.04, *p* <  0.02; beta coefficient = − 0.03, *p* <  0.02). None of the stress-related biomarkers showed a statistically significant association with abdominal pain severity. Each of the stress-related perceptions—*perceived stress, tension,* and *anxiety*—was significantly related to higher abdominal pain severity (beta coefficient = 0.009, *p* <  0.06; beta coefficient = 0.05, *p* <  0.001; beta coefficient = 0.06, *p* <  0.001; see Table 2).

**Table 2:** Univariate random-effects models for abdominal pain severity (β_1_) with age as predictor (β_2_) and with covariates (β_3_) individually entered

	Mean Values			Standard Deviations			Number	
	(*p* - *values*)							
	Intercept	Slope	Covariate	ntercept	Slope	Residual Error		
Predictor	β_1_^a^	β_2_^a^	β_3_^a^	σ_1_^b^	σ_2_^b^	σ_3_^b^	Women	Observations
Age (47.6 years)	0.23	−.003 (0.220)	–	0.29	0.02	0.36	291	6977
Reproductive Aging Markers								
Late Reproductive ^c^	0.24 (< 0.001)	−0.001 (0.750)		0.29	0.02	0.36	291	6977
Early MT			− 0.001 (0.970)					
Late MT			−0.007 (0.780)					
Early PM			− 0.04 (0.160)					
Urinary Estrone (ng/mg creatinine, Log_10_)	0.20 (< 0.001)	− 0.01 (< 0.001)	− 0.04 (0.020)	0.28	0.01	0.35	131	4908
Urinary FSH (mIU/mg creatintine, Log_10_)	0.20 (< 0.001)	−0.01 (< 0.001)	− 0.004 (0.720)	0.28	0.02	0.35	131	4996
Urinary Testosterone (ng/mg creatinine, Log_10_)	0.20 (< 0.001)	−0.01 (< 0.001)	− 0.03 (0.020)	0.28	0.02	0.35	131	4975
Stress-related Biomarkers								
Urinary Cortisol (ng/mg creatinine, Log_10_)	0.20 (< 0.001)	−0.01 (< 0.001)	− 0.01 (0.310)	0.28	0.02	0.35	131	4993
Urinary Epinephrine (ng/mg creatinine, Log_10_)	0.20 (< 0.001)	−0.01 (< 0.001)	0.0004 (0.940)	0.28	0.01	0.35	130	3325
Urinary Norepinephrine (ng/mg creatinine, Log_10_)	0.20 (< 0.001)	−0.01 (< 0.001)	0.005 (0.820)	0.27	0.01	0.35	130	3329
Stress-related Perceptions								
Perceived Stress (1–6)	0.21 (< 0.001)	− 0.003 (0.280)	0.009 (0.060)	0.29	0.02	0.36	291	6977
Tension (0–4)	0.20 (< 0.001)	−0.003 (0.190)	0.05 (< 0.001)	0.28	0.02	0.36	291	6977
Anxiety (0–4)	0.19 (< 0.001)	−0.003 (0.190)	0.06 (< 0.001)	0.27	0.02	0.36	291	6977

^a^ β_1,_ β_2,_ β_3_ are the fixed effects (group averages) for the intercept, slope and covariate, respectively. β_1_ represents the mean value in abdominal pain severity for all women in the sample at the mean centered age (47.6 years); β_2_ represents the rate and direction (+ or -) of change in abdominal pain severity per year; and β_3_ represents the change in mean abdominal pain severity score for every unit of change in covariate score, when the covariate is added to the model

^b^ σ_1,_ σ_2,_ σ_3_ are the random effects (variability) for the intercept, slope and residual error, respectively

^c^ Reference group for this categorical variable

The statistically significant covariates from the univariate analysis, as well as *age* (as the measure of time), were then included in a multi-variate random effects model (E_1_G, testosterone, perceived stress, tension, and anxiety). Statistical significance was set as *p* <  0.05. When *perceived stress*, *anxiety*, and *tension* were included together in the final model (Table 3), the effect of tension was not statistically significant and the beta coefficient for perceived stress changed from positive in the univariate model to negative, suggesting multicollinearity. We removed both stress and tension from subsequent models. Additionally, when *testosterone* was included with *E*_*1*_*G* in the model, neither predictor met our criterion for statistical significance. We then tested a final model with *age*, *anxiety*, and *E*_*1*_*G*. When *E*_*1*_*G* and *testosterone* were analyzed separately with *anxiety*, both were significant (*p* = 0.04). The effect of *E*_*1*_*G* was greater than that of *testosterone* (beta coefficient = − 0.04 for *E*_*1*_*G* vs. beta coefficient = − 0.03 for testosterone), but the difference of the magnitude of the effect was not large. As seen in the model tested in Table 4, *age* was significantly associated with lower abdominal pain severity (beta coefficient = − 0.01, *p* <  0.001) and *anxiety* was significantly associated with greater abdominal pain severity (beta coefficient = 0.06, *p* = 0.00). *E*_*1*_*G* was significantly associated with lower abdominal pain severity (beta coefficient = − 0.04, *p* <  0.04) in this model.

**Table 3:** Preliminary multivariate mixed-effects model for abdominal pain severity with age as predictor and significant covariates simultaneously entered (*n* = 131; observations = 4890)

	Beta Coefficient ^a^	Standard Error/Standard Deviation	*P*-value
Fixed effects			
β_1_ Intercept	0.18	0.03	< 0.001
β_2_ Age (−47.6) years	−0.01	0.003	< 0.001
β_3_ Perceived Stress	−0.01	0.007	0.040
β_4_ Urinary Estrone (Log ^10^)	−0.03	0.02	0.090
β_5_ Urinary Testosterone (Log ^10^)	−0.02	0.01	0.080
β_6_ Tension	0.002	0.01	0.830
β_7_ Anxiety	0.06	0.01	< 0.001
Random effects			
b_1_ Intercept σ_1_		0.26	
b_2_ Age (−47.6) years σ_2_		0.01	
b_3_ Residual σ_3_		0.35	

^a^ The beta coefficient is a measure of the change in abdominal pain severity for every one unit of change in each respective predictor variable

**Table 4:** Final multivariate mixed-effects model for abdominal pain severity with age as predictor and significant covariates simultaneously entered (n = 131; observations = 4890)

	Beta Coefficient ^a^	Standard Error/Standard Deviation	*P-*value
Fixed effects			
β_1_ Intercept	0.16	0.02	< 0.001
β_2_ Age (−47.6) years	−0.01	0.003	0.001
β_3_ Urinary Estrone (Log^10^)	−0.04	0.02	0.040
β_4_ Anxiety	0.05	0.01	< 0.001
Random effects			
b_1_ Intercept σ_1_		0.26	
b_2_ Age (−47.6) years σ_2_		0.02	
b_3_ Residual σ_3_		0.35	

^a^ The beta coefficient is a measure of the change in abdominal pain severity for every one unit of change in each respective predictor variable

## Discussion

The results presented here are the first reported on abdominal pain experienced during the MT and early PM captured in a longitudinal study, the analysis of which involved multiple repeated measures of symptom severity, MT stages, reproductive hormone biomarkers, stress-related biomarkers, and stress-related perceptions. Analyses reported here reveal that factors associated with abdominal pain change as a woman ages through the MT.

In the univariate analyses—in which each covariate was independently analyzed for an association with abdominal pain—neither *age* nor any of the MT stages was found to be a significant predictor of abdominal pain, nor were any of the stress-related biomarkers or the reproductive biomarker *FSH*. *E*_*1*_*G* and *testosterone* were both significant predictors of lower abdominal pain severity and all three stress-related perceptions were significant predictors of higher abdominal pain severity in the univariate analysis. Only *anxiety* was included in the final multivariate analysis due to multicollinearity with perceived stress. Both *E*_*1*_*G* and *testosterone* did not meet significance criteria when included in the model, but *E*_*1*_*G* alone had a significant effect when included with *age* and *anxiety*. In the multivariate analysis, *age* and *E*_*1*_*G* were associated with lower abdominal pain severity and *anxiety* with greater abdominal pain severity.

Abdominal pain prevalence has been reported to decrease in association with advancing age [7, 8]. Our findings are consistent with this, suggesting that abdominal pain will become less severe throughout the MT and into PM as a consequence of aging. In addition to age, it is possible that this decrease is also due to the cessation of menstruation, and thus, the dysmenorrhea that many women experience during the late reproductive and menopausal transition years [36]. The Study of Women’s Health Across the Nation (SWAN)—a multi-site, prospective observational cohort of 3297 community-based women, aged 42–52, followed from 1996 to 2011—found that women who reported abdominal cramps from menses during their reproductive years had the largest decreases in overall body pain as they transitioned through menopause and into their PM years, suggesting that this decrease may be due to the resolution of dysmenorrhea [11]. The similarity between the lower abdominal pain reported in this current study and the decreases seen in the SWAN study are valuable findings that may give insight into abdominal pain in the MT and PM.

Also consistent with the current literature was the association found in this study between anxiety and higher abdominal pain. In a study assessing the relationships between anxiety, depression, and abdominal pain in a general adult population, Walter et al found that higher anxiety scores were associated with a higher prevalence of abdominal pain, as well as a higher pain score [37]. Participants with higher anxiety scores also reported more abdominal pain episodes per week, and longer duration of pain in hours; these results were greater in women compared to men. Such results suggest that there may be a causal link between anxiety in women and abdominal pain, further supported by research that has found more somatoform symptoms, a higher lifetime rate of anxiety disorders, and a higher prevalence of IBS in women compared to men [38–40]. Additionally, a systematic review on the impact of attitude towards menopause on symptom experience concluded that women with negative attitudes towards menopause report more symptoms during the MT. [41] Yet another study, a cross-sectional study of 992 community-based women assessing the perceived impact of life events on symptoms experienced throughout the MT and PM, gathered questionnaire data and found that life events significantly predicted all menopausal symptoms (physical and psychological) with the exception of urinary symptoms [42]. These results suggest that much of the symptomology experienced throughout the MT and PM may be impacted by factors such as response to stressful events and anticipation of menopause. However, while this association has been identified in several studies, none have been specifically in an MT and PM population. Thus, these results contribute new findings to the existing body of literature and prompt future study in an MT and early PM population.

Regarding reproductive biomarkers, our results suggest that both *E*_*1*_*G* and *testosterone* contribute to abdominal pain severity, but when effects of both are compared, *E*_*1*_*G* levels had a greater negative association with abdominal pain severity in MT and PM women. Moreover, *age* had a negative association with abdominal pain severity, possibly attributable to declining levels of estrogen as women move from late reproductive to postmenopause stages. It is well-documented that, compared to men, women exhibit greater pain sensitivity, reduced pain inhibition, enhanced pain facilitation, more pain-related conditions, and an increased risk for clinical pain [43–45]. Combined with the clear and well-documented greater prevalence of pain in women compared to men—including abdominal pain—our results suggest a sex-specific mechanism of action for pain in the MT and PM [8].

The lack of significant association between *testosterone* with abdominal pain when *E*_*1*_*G* was included in the final analytical model is consistent with previously published results from the SMWHS that found no association between several different pain measures and testosterone; unlike the current study, however, the same was found to be true of E_1_G in relation to the pain measures in the prior SMWHS report [13]. These conflicting results regarding E_1_G are mirrored in several other studies. In addition to the previous SMWHS findings, two large cohort studies reported similar results. The Melbourne Women’s Midlife Health Project (MWMHP) and the SWAN found that while bodily pain increased throughout each stage of the MT and into the early PM years, these increases occurred independent of reproductive hormones [13, 46, 47]. In contrast, Nikolov and Petkova designed a cohort study to investigate the influence of estrogen on pain sensitivity in menopausal women with low back pain and found a significant association (*p* <  0.0005) between decreasing estrogen levels and pain intensity [48]. Interestingly, Nikolov and Petkova found *similar* results to the MWMHP and the SWAN studies regarding pain and menopausal status—that pain intensity was significantly associated with menopausal status (*p* <  0.002), even after adjusting for all other variables (*p* <  0.001). When considered together with results from the current study, it is clear that more research is necessary to understand the complex interplay of reproductive hormones, pain, and the MT. What we definitively know is that estrogens help to regulate and modulate the opioid system, contributing to varying levels of anti- and nociception [49–54]. We do not yet know how this regulation affects abdominal pain experienced in the MT. Both the experience of pain and the MT are biopsychosocial in nature, and so it is likely that many more factors beyond estrogen level, as well as beyond the scope of the current study, contribute to the experience of pain in the MT and PM. While the current study corroborates the pivotal role of estrogen in pain regulation during the MT and PM, future explorations of the possible impact biopsychosocial factors may have on them will lend clarity to the experience of pain in the MT and PM. Studying larger numbers of women during both the MT and early PM may help to clarify the relationship between progression through the stages of reproductive aging and abdominal pain, as well as measuring a broader spectrum of reproductive biomarkers throughout this transition.

### Strengths and limitations

The primary merit of the SMWHS was its longitudinal nature, which provided a large set of MT-stage-anchored data that allowed abdominal pain to be analyzed not only in terms of covariate associations, but also for the possible change in those associations over time and in relation to the stage of MT in which they were experienced. These analyses are invaluable in the realm of MT research, as they have not been done prior to the current study and are important in order to advance our knowledge on abdominal pain experienced throughout the MT and beyond.

When interpreting the results of this study, four limitations should be taken into consideration. First, population characteristics differed significantly in family income, years of education, and race/ethnicity (see Table 1). Over the course of the 23 years of data collection, non-white ethnic participants, as well as participants from a lower socioeconomic status, were more likely to exit the study. This may limit the generalizability of these findings to some women. Secondly, the study population was of modest size, which could have masked associations between covariates and symptom severity. It may be the case that these associations would be revealed by a larger sample size. Despite this, the study contributed significantly to our understanding of the relationships studied over the MT and early PM, as large numbers of repeated measures were amassed from the albeit modest sample size, resulting in a robust repository of data. Thirdly, while only healthy participants were recruited, new diagnoses throughout the study were not cause for discontinuation of study participation, nor was the development of such diagnoses tracked during the follow-up period. Abdominal pain in women of all ages may be attributed to many different pathologies—such as gastrointestinal and gynecological disorders—and increases in abdominal pain can be associated with increases in pathology-specific pain [16, 55]. It was beyond the scope of this study to distinguish between primary abdominal pain and secondary or referred abdominal pain, and as such, associations between covariates and abdominal pain severity could have been impacted. Fourth, while we did not see an association between *MT Stage* and abdominal pain severity, we did see an association between *age* and abdominal pain severity. It is possible that this lack of association with the MT stages is due to collinearity between *age* and *MT Stage*. Future studies would benefit from exploring further the possible interplay between these variables.

## Conclusion

In summary, abdominal pain experienced in MT and early PM women is lower throughout the MT and into the PM, as associated with increasing age. This association of lower pain with increasing age is consistent with the literature. Although it is clear that anxiety is associated with higher abdominal pain, the role of perceived stress on abdominal pain in the MT and PM remains worthy of additional exploration. The reproductive biomarkers E_1_G and testosterone are associated with lower abdominal pain when considered individually; however, more research is necessary to determine if this association remains when other factors are considered. When working with women experiencing abdominal pain during the MT or early PM, clinicians should keep in mind that biological as well as psychosocial factors may be contributing to the severity of their pain. As the first reported longitudinal study of abdominal pain experienced by women during the MT and early PM to be published, the findings reported here suggest relationships between age, reproductive biomarkers, stress-related perceptions, and symptom severity that warrant further exploration.

## Data Availability

Although analyses are still in progress from the data, our intention is to make the database available to other investigators.

## Abbreviations

AIC: Akaike information criterion
E1G: Estrone glucuronide
ED: Emergency department
FMP: Final menstrual period
FSH: Follicle stimulating hormone
GI: Gastrointestinal
IBS: Irritable bowel syndrome
MLM: Multi-level modeling
MT: Menopause transition
MWMHP: Melbourne Women’s Midlife Health Project
PM: Early postmenopause
QOL: Quality of life
SD: Standard deviation
SMWHS: Seattle Midlife Women’s Health Study
STRAW: Stages of reproductive aging workshop
SWAN: Study of Women’s Health Across the Nation
US: United States
